# Autoantibodies to Estrogen Receptor α in Systemic Sclerosis (SSc) as Pathogenetic Determinants and Markers of Progression

**DOI:** 10.1371/journal.pone.0074332

**Published:** 2013-09-18

**Authors:** Antonello Giovannetti, Angela Maselli, Tania Colasanti, Edoardo Rosato, Felice Salsano, Simonetta Pisarri, Ivano Mezzaroma, Walter Malorni, Elena Ortona, Marina Pierdominici

**Affiliations:** 1 Department of Clinical Medicine, Division of Clinical Immunology, “Sapienza” University, Rome, Italy; 2 Department of Cell Biology and Neurosciences, Istituto Superiore di Sanità, Rome, Italy; 3 IRCCS San Raffaele Pisana, Rome, Italy; 4 Department of Therapeutic Research and Medicines Evaluation, Istituto Superiore di Sanità, Rome, Italy; 5 San Raffaele Pisana, L’Aquila, Italy; University of Pittsburgh, United States of America

## Abstract

Systemic sclerosis (SSc) is a multisystem autoimmune disease of unknown etiology characterized by inflammation, autoantibody production, and fibrosis. It predominantly affects women, this suggesting that female sex hormones such as estrogens may play a role in disease pathogenesis. However, up to date, the role of estrogens in SSc has been scarcely explored. The activity of estrogens is mediated either by transcription activity of the intracellular estrogen receptors (ER), ERα and ERβ, or by membrane-associated ER. Since the presence of autoantibodies to ERα and their role as estrogen agonists interfering with T lymphocyte homeostasis were demonstrated in other autoimmune diseases, we wanted to ascertain whether anti-ERα antibodies were detectable in sera from patients with SSc. We detected anti-ERα antibody serum immunoreactivity in 42% of patients with SSc (30 out of 71 analyzed). Importantly, a significant association was found between anti-ERα antibody values and key clinical parameters of disease activity and severity. Fittingly, anti-ERα antibody levels were also significantly associated with alterations of immunological features of SSc patients, including increased T cell apoptotic susceptibility and changes in T regulatory cells (Treg) homeostasis. In particular, the percentage of activated Treg (CD4^+^CD45RA^−^ FoxP3^bright^CD25^bright^) was significantly higher in anti-ERα antibody positive patients than in anti-ERα antibody negative patients. Taken together our data clearly indicate that anti-ERα antibodies, probably *via* the involvement of membrane-associated ER, can represent: i) promising markers for SSc progression but, also, ii) functional modulators of the SSc patients’ immune system.

## Introduction

Estrogens are well-known regulators of the immune responses and several lines of evidence support a key role for them in the development or progression of numerous diseases, including autoimmune disorders [1]–[5]. Estrogens, in particular 17β-estradiol, directly modulate the function of immune cells by transcriptional activity of nuclear estrogen receptors (ER), i.e., ERα and ERβ [1]. Recently, the expression of functional membrane-associated ERα in different cell types including human lymphocytes has been suggested [6]–[8] and autoantibodies specific to ERα have been detected in sera from patients with systemic lupus erythematosus (SLE) [9]. These anti-ERα antibodies behave as true estrogen agonist and are able to induce cell activation and apoptotic cell death in resting lymphocytes as well as proliferation of anti-CD3 activated T cells. Interestingly, a significant association between anti-ERα antibody titer and disease activity was demonstrated [9]. Similarly to SLE, systemic sclerosis (SSc) is an autoimmune disease characterized by multiorgan involvement and circulating autoantibodies against intracellular antigens [10]. The pathogenesis of SSc is complex and incompletely understood. Immune activation, vascular damage, and connective tissue fibrosis are all known to be important in the development of this disease [11], [12]. Overall, a substantial female predominance exists in SSc, with a female-to-male ratio ranging from 3∶1 to 14∶1 [10], suggesting that female sex hormones such as estrogen may play a role in disease pathogenesis. Nevertheless, only limited information is currently available on the role of estrogens in SSc [13]–[15] and the presence of anti-ERα antibodies has not been explored yet. Therefore, the aim of this study was to evaluate anti-ERα serum immunoreactivity in patients with SSc and to assess the possible relationship between the presence of anti-ERα antibodies and the clinical and immunological features of the disease.

## Materials and Methods

### Ethics Statement

This study has been conducted according to the principles expressed in the Declaration of Helsinki. Written informed consent was obtained from all patients and controls, and the study was approved by the Ethical Committee of “Policlinico Umberto I”, Rome, Italy.

### Patients and Biological Samples

We analyzed sera from 71 consecutive patients with SSc (**Table 1**). All patients fulfilled the preliminary criteria for SSc as defined by the American College of Rheumatology [16]. SSc was diffuse (dcSSc) in 29 patients and limited (lcSSc) in 42 patients. Disease activity was evaluated using the European Scleroderma Study Group (EScSG) activity index [17]. All 71 patients had anti-nuclear antibody (ANA) (indirect immunofluorescence on Hep-2000 cells), 24 out of 29 dcSSc patients had anti-topoisomerase I antibodies (anti-Scl70 antibodies) and 17 out of 41 lcSSc patients had anti-centromere (ACA) antibodies (Innogenetics, Gent, Belgium). All patients underwent nailfold capillaroscopy and were divided in three different capillaroscopic patterns: early, active and late [18]. Carbon monoxide diffusion capacity (DLCO) was measured by the single breath method, according to the American Thoracic Society standards [19]. Exclusion criteria were previous or concomitant treatments with immunosuppressive drugs. Twenty-two (31%) patients were on low dose steroids (below 10 mg of prednisone a day) at the time of inclusion in the study. The control group consisted of 90 healthy donors matched for age and sex with the SSc group. For flow cytometry analysis, 34 out of 90 healthy donors were randomly selected as representative of the whole series.

**Table 1:** Demographic and clinical characteristics of SSc patients (n = 71).

Age, median (range) years	56 (21–79)
Sex, n. of men/n. of women	11/60
Disease duration, median (range) years	8 (1–37)
Disease type (dcSSc/lcSSc)	29/42
EScSG, mean (±SD)	2 (1.6)
DLCO, mean (±SD), % of predicted value	73.2 (19.7)
NC pattern:	
Early	18 (25)
Active	18 (25)
Late	35 (49)
ACA	19 (27)
Scl70	30 (42)
Steroid treatment*	22 (31)

Except where indicated otherwise, values are the absolute number and the percentage (in brackets) of patients.

* Steroid treatment during the last 6 months. n., number; dcSSc, diffuse cutaneous systemic sclerosis; lcSSc, limited cutaneous systemic sclerosis; EScSG, European Scleroderma Study Group activity index; DLCO, carbon monoxide lung diffusion capacity; NC, nailfold capillaroscopy; ACA, anti-centromere antibody; Scl70, anti-topoisomerase I antibody.

### Enzyme-Linked Immunosorbent Assay (ELISA)

We analyzed serum IgG immunoreactivity to ERα by ELISA in patients with SSc and in healthy donors. ELISA was developed as previously described [9]. Briefly, polystyrene plates (Maxisorp, Nunc, Roskilde, Denmark) were coated with the antigen (2 µg/well ERα, Sigma, St Louis, MO, USA) in 0.05 M NaHCO3 buffer, pH 9.5, and incubated overnight at 4°C. Plates were blocked with 100 µl/well of 3% milk for 1 hour at 37°C. Human sera were diluted 1∶100 in phosphate buffered saline (PBS)-Tween (PBST) and 1% milk, 100 µl per well. Peroxidase-conjugated goat anti-human IgG (BioRad, Richmond, CA, USA) were diluted in PBST containing 1% milk (1∶3,000) and incubated for 1 hour at room temperature. *O*-phenylenediamine dihydrochloride (Sigma) was used as a substrate and the optical density (OD) was measured at 490 nm (OD_490_). Three SD above the mean OD reading in the healthy donors was considered the cutoff level for positive reactions. All assays were performed in quadruplicate. Data are presented as the mean OD corrected for background (wells without coated antigen). The results of unknown samples on the plate were accepted if internal controls (2 serum samples, 1 positive and 1 negative) had an absorbance reading within mean ±10% of previous readings. To evaluate the antibody specificity, the serum pool obtained from patients with SSc who were positive for anti-ERα antibodies was diluted 1∶100 in PBST and incubated overnight at room temperature in the presence of 40 µg/ml of ERα (Sigma) or, as a negative control, with 40 µg/ml of bovine serum albumin (BSA; Sigma).

### Cell Isolation and Flow Cytometry

For flow cytometry analyses, blood samples from 34 patients with SSc and an equal number of healthy donors were studied. Peripheral blood mononuclear cells were isolated by a Ficoll-Hypaque density-gradient separation (Lympholyte-H; Cedarlane Laboratories, Hornby, Ontario, Canada) and spontaneous apoptosis was measured immediately after separation (*ex vivo* apoptosis) as described below. Cell surface and intracellular phenotyping were performed with combinations of monoclonal antibodies (mAb) conjugated with fluorescein isothiocyanate (FITC), phycoerythrin (PE), peridinin chlorophyll protein, or allophycocyanin, as previously described [20]. Conjugated mAb against human CD3, CD4, CD8, CD45RA, CD62L, CD25, CD95, HLA-DR (all from BD Immunocytometry Systems, San Jose, CA, USA), and Bcl-2 (DAKO, Glostrup, Denmark) were used. The naive subset was defined as CD45RA^+^CD62L^+^ while the remaining cells comprised the memory subsets (CD45RA^−^CD62L^+^, central memory subset; CD45RA^−^CD62L^−^ and CD45RA^+^CD62L^−^, effector memory subset) [20]. For detection of regulatory T cells (Treg, CD4^+^CD25^+^FoxP3^+^), intracellular detection of FoxP3 with Alexa Fluor® 488-conjugated anti-FoxP3 (Clone 259D, Biolegend, San Diego, CA, USA) was performed on fixed and permeabilized cells according to the manufacturer’s protocol (Biolegend). Activated CD4^+^ Treg (aTreg) was defined as CD4^+^CD45RA^−^ FoxP3^bright^CD25^bright^ [21].

Apoptosis was quantified using a FITC-conjugated annexin V and propidium iodide apoptosis detection kit (Marine Biological Laboratory, Woods Hole, MA, USA) according to the manufacturer’s protocol. Reported data are referred to annexin V-positive apoptotic cells.

Acquisition was performed on a FACSCalibur flow cytometer (BD Immunocytometry Systems) and data were analyzed using the Cell Quest Pro software (BD Immunocytometry Systems).

### Statistical Analyses

The results are expressed as mean ± standard deviation (SD), unless otherwise indicated. For group comparisons of continuous variables, the Mann-Whitney U-test or the Kruskal Wallis test and Dunn’s multiple comparison test were used. For categorical variables, chi-square test was used. Correlations were evaluated by using Spearman’s rank correlation test. Linear regression analysis was used to display a best fit line to the data. Statistical analyses were performed using GraphPad Prism, version 5.0 software (GraphPad Software, San Diego, CA, USA). All tests were 2-sided and a *P* value<0.05 was considered statistically significant.

## Results

### Serum Anti-ERα Antibodies in Patients with SSc

**Figure 1:**
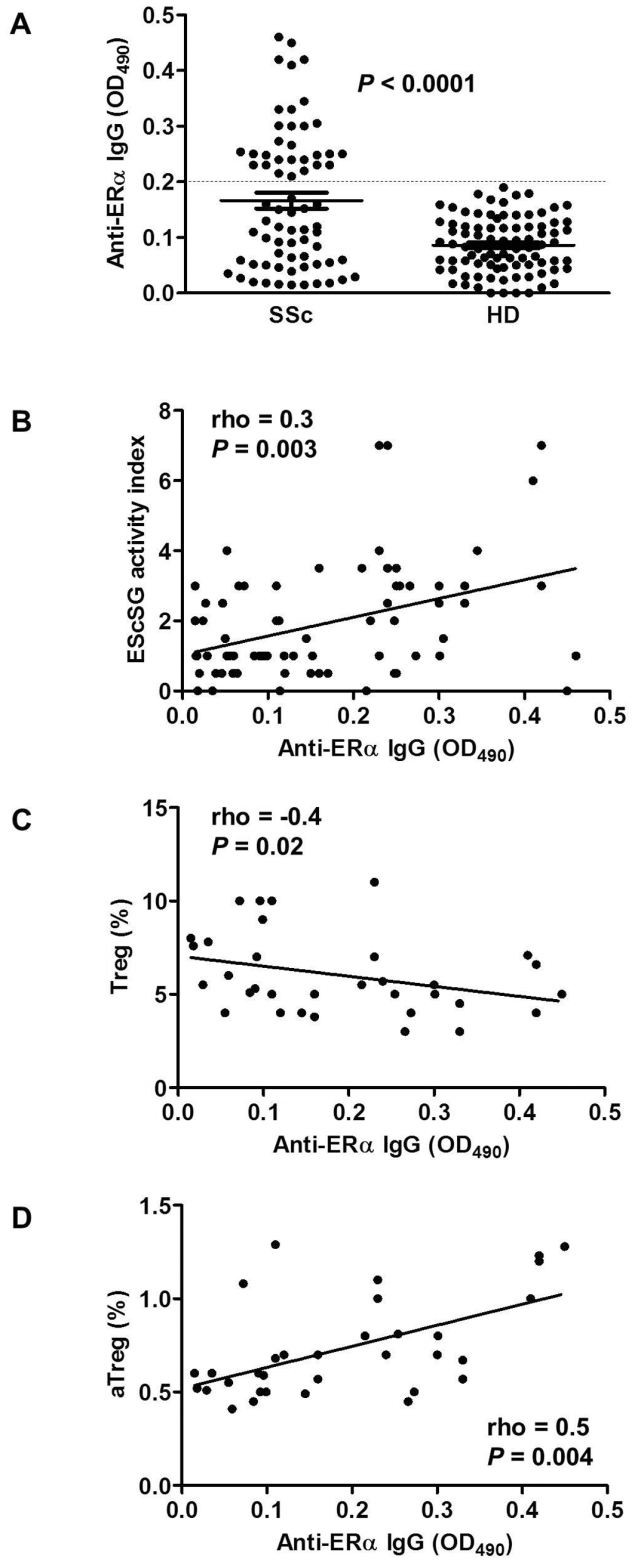
Evaluation of serum anti-ERα antibody titer in patients with SSc and healthy donors. (**A**) Anti-ERα antibodies in SSc patients (n = 71) and in sex- and age-matched healthy donors (HD, n = 90). Samples were considered positive if the optical density (OD) at 490 nm was higher than the cutoff value of an OD at 490 nm of 0.2 (broken line). The cut-off value was defined as 3 SD above the mean OD at 490 nm in healthy donors. Circles represent individual samples. *P*<0.0001 mean OD at 490 nm in patients with SSc *vs*. healthy donors (Mann-Whitney U-test). (**B**) Correlation between anti-ERα antibody levels and the EScSG activity index in SSc (n = 71). (**C**) Correlation between anti-ERα antibody levels and the percentage of circulating Treg (CD4^+^CD25^+^FoxP3^+^) and (**D**) that of activated Treg (aTreg, CD4^+^CD45RA^−^ FoxP3^bright^CD25^bright^) in SSc (n = 34). The rho and *P* values shown in panels B–D were determined using the Spearman’s rank correlation analysis. Solid lines represent best fits as estimated by linear regression analysis.

We detected anti-ERα antibodies in 30/71 (42%) patients with SSc whereas no anti-ERα antibodies were found in sera from healthy donors (**Figure 1A**). Preabsorption of the pooled positive sera (from 30 patients with SSc) with ERα completely inhibited the antibody immunoreactivity, thus confirming the specificity of ELISA (data not shown).

### Association between Anti-ERα Antibodies and Clinical Variables

In order to evaluate whether the presence of anti-ERα antibodies plays a role in the clinical course of the disease, we divided SSc patients into subgroups according to the presence of serum anti-ERα antibodies and examined the demographic and clinical parameters in each group. Only significant results are reported in **Table 2**. The presence of serum anti-ERα antibodies was significantly associated with the diffuse form of the disease (*P* = 0.02), the presence of anti-Scl70 antibodies (*P* = 0.03), the EScSG activity index (*P* = 0.001) and the late capillaroscopic pattern (*P* = 0.04). Spearman’s rank analysis also showed a significant positive correlation between the anti-ERα antibody level and the EScSG activity index (Spearman’s rho* = *0.3, *P* = 0.003), **Figure 1B**. No further significant association was found with the demographic and clinical parameters evaluated in the present study nor with the different pharmacological treatments.

**Table 2:** Clinical characteristics of SSc patients (n = 71) divided according to the presence or absence of serum anti-ERα antibodies.

Clinical characteristics*	Anti-ERα IgG-positive (n = 30)	Anti-ERα IgG-negative (n = 41)	*P* values
Disease type (dcSSc/lcSSc)	17/13	12/29	0.02
EScSG activity index (mean ± SD)	2.7±2	1.3±1	0.001
NC:			
early	4 (13)	14 (34)	>0.05
active	7 (23)	11 (27)	>0.05
late	19 (63)	16 (39)	0.04
Scl70	17 (57)	13 (32)	0.03

* Except where indicated otherwise, values are the absolute number and the percentage (in brackets) of patients. Statistical analyses were performed using Mann-Whitney U-test for EScSG and the chi-square test for all other parameters. dcSSc, diffuse cutaneous systemic sclerosis; lcSSc, limited cutaneous systemic sclerosis; EScSG, European Scleroderma Study Group activity index; NC, nailfold capillaroscopy; Scl70, anti-topoisomerase I antibody.

### Association between Anti-ERα Antibodies and Immunological Parameters

We also evaluated the association between the presence of serum anti-ERα antibodies and different immunological parameters including the peripheral distribution of T lymphocyte subsets (naive, central and effector memory lymphocytes and Treg), the expression of relevant markers associated with cell activation and cell fate (i.e., HLA-DR, CD95, Bcl-2) and the level of *ex vivo* apoptosis. To this aim, 34 patients, randomly selected as representative of the whole series, were studied. The demographic characteristics and the immunophenotypic profile of this patient population, divided into subgroups according to the presence or absence of serum anti-ERα antibodies (anti-ERα IgG-positive, n = 16 and anti-ERα IgG-negative, n = 18), were shown in **Table 3**. The proportion of CD4^+^CD25^+^FoxP3^+^ Treg cells was lower in anti-ERα antibody positive patients than in anti-ERα antibody negative patients (5.2±2% and 7.2±2%, respectively, *P*<0.05). A negative correlation between anti-ERα antibody levels and frequencies of CD4^+^CD25^+^FoxP3^+^ Treg was also found (Spearman’s rho = −0.4, *P* = 0.02; **Figure 1C**). On the contrary, the proportion of CD4^+^CD45RA^−^ FoxP3^bright^CD25^bright^ aTreg was significantly higher in anti-ERα antibody positive patients than in anti-ERα antibody negative patients (0.85±0.3% *vs.* 0.63±0.2%, respectively, *P*<0.05). Moreover, the Spearman’s rank analysis showed a significant positive correlation between the proportion of aTreg and the anti-ERα antibody level (Spearman’s rho = 0.5, *P* = 0.004; **Figure 1D**). Significant differences between anti-ERα antibody positive and anti-ERα antibody negative patients were also found for CD4^+^CD95^+^ frequencies (55±13% *vs.* 44±12%, respectively, *P*<0.05), *ex vivo* apoptosis level (10±4% *vs.* 7.2±3%, respectively, *P*<0.05), and Bcl-2 expression (mean fluorescence intensity calculated as the ratio between Bcl-2 mAb mean and negative control mean, 22±6 *vs.* 28±6, respectively, *P*<0.05). The Spearman’s rank analysis showed a significant positive correlation between anti-ERα antibody level and CD4^+^CD95^+^ frequencies (Spearman’s rho = 0.35, *P* = 0.04) and a significant negative correlation for anti-ERα antibody level and Bcl-2 expression (Spearman’s rho = −0.35, *P* = 0.04).

**Table 3:** Demographic characteristics and immunophenotypic profile of randomly selected SSc patients, divided according to the presence or absence of serum anti-ERα antibodies, and healthy donors.

Patients’ features	Anti-ERα IgG-positive(n = 16)	Anti-ERα IgG-negative(n = 18)	Healthy donors(n = 34)	*P* values
**Demographic characteristics**				
Age, median (range) years	55 (21–74)	59 (35–79)	57 (26–75)	>0.05
Sex, n. of men/n. of women	2/14	3/15	5/29	>0.05
**Immunophenotypic profile**				
CD3^+^CD4^+^ (%)	49±9	53±9	47±6	>0.05
CD3^+^CD4^+^ naive (%)	47±11	53±12	47±12	>0.05
CD3^+^CD4^+^ central memory (%)	40±9	39±9	39±8	>0.05
CD3^+^CD4^+^ effector memory (%)	13±9	8±5	11±6	>0.05
CD3^+^CD4^+^HLA-DR^+^ (%)	7±7	4±3	4±2	>0.05
CD3^+^CD4^+^CD95^+^ (%)	55±13*	44±12	47±9	0.03
Treg (%)	5.2±2*	7.2±2**	4.5±1.5	0.0001
aTreg (%)	0.85±0.3*,**	0.63±0.2	0.58±0.3	0.007
CD3^+^CD8^+^ (%)	22±7	17±8	21±6	>0.05
CD3^+^CD8^+^ naive (%)	46±19	51±15	50±17	>0.05
CD3^+^CD8^+^ central memory (%)	14±7	17±8	17±8	>0.05
CD3^+^CD8^+^ effector memory (%)	39±19	31±12	30±10	>0.05
CD3^+^CD8^+^HLA-DR^+^ (%)	17±13	16±15	13±9	>0.05
CD3^+^CD8^+^CD95^+^ (%)	67±21	63±19	53±17	>0.05
*Ex vivo* lymphocyte apoptosis (%)	10±4*,**	7.2±3	6.7±2.5	0.02
Lymphocyte Bcl-2 expression (MFI)	22±6*	28±6	24±6	0.01

n., number. For CD4^+^ and CD8^+^ lymphocyte subsets, data were expressed as the percentage of each subset within the CD4^+^ or CD8^+^ population considered as 100%. The naive subset is defined as CD45RA^+^CD62L^+^ while the remaining cells comprise the memory subsets (CD45RA^−^CD62L^+^, central memory subset; CD45RA^−^CD62L^−^ and CD45RA^+^CD62L^−^, effector memory subset) [20]. T regulatory cells (Treg) are defined as CD4^+^CD25^+^FoxP3^+^; activated Treg (aTreg) are defined as CD4^+^CD45RA^−^FoxP3^bright^CD25^bright^ [21]. Mean fluorescence intensity (MFI) was calculated as the ratio between Bcl-2 mAb mean and negative control mean. The Kruskal Wallis test and Dunn’s multiple comparison test were used for statistical analysis.

* *P*<0.05 between the two groups of patients,

** *P*<0.05 *vs*. healthy donors.

## Discussion

In this study, we demonstrated for the first time the presence of serum anti-ERα antibodies in SSc. Anti-ERα antibodies were significantly associated with disease activity and were mainly found among patients with the diffuse form of the disease, the Scl70 positivity, and the late capillaroscopy pattern. Anti-ERα antibody positivity was also associated with diverse alterations of immunological features, including increased T cell apoptotic susceptibility and a higher percentage of aTreg.

Several lines of evidence suggest a hierarchical predominant role of the T cell-mediated immune response in inducing SSc [11], [22], [23] and an altered T cell homeostasis has been associated with a more severe disease course and phenotype [24]. Here, we found that anti-ERα antibody levels negatively correlated with the peripheral frequency of Treg. This finding is consistent with the critical role of this cell subset in maintaining self tolerance and preventing autoimmunity [25]. Interestingly, different subsets of Treg, displaying unique functional and homeostatic properties, have been recently described [21]. Notably, in this study, we found a significantly higher level of circulating aTreg in anti-ERα antibody positive SSc patients than in anti-ERα antibody negative patients. In the same vein, Mathian and coworkers [23] found higher levels of aTreg in SSc patients with the diffuse and/or active disease (here demonstrated to be also, in large part, anti-ERα positive) suggesting a compensatory, but inefficient, amplification of regulatory cells in the context of active inflammation. However, one recent report demonstrated contrary results, showing lower aTreg levels in SSc patients than in healthy donors although in this case the variable “disease form” (i.e., diffuse and limited) was not considered [26]. Conflicting results have been also reported for other autoimmune diseases such as SLE in which both normal [27] or decreased [21] levels of aTreg have been detected. Variables such as genetic background or different criteria for patient selection (e.g., disease stage, clinical form, duration, and treatment) possibly contribute to these contrasting results impairing a comparative analysis of aTreg in different reports, and further studies are needed to better address the distribution and the role of Treg subpopulations in autoimmune diseases.

Anti-ERα antibody positivity was also significantly associated with T cell apoptosis and apoptotic related molecules. This finding is in line with our previous *in vitro* data on the ability of the anti-ERα antibodies to induce apoptosis in T lymphocytes from healthy donors [9]. Apoptosis plays an important role in bypassing tolerance to intracellular autoantigens [28]. Increased apoptosis detected in autoimmune diseases, such as SSc [24], may lead to an autoantigen overload, initiating and/or perpetuating an autoimmune response and an autoantibody-mediated tissue damage [28]. Apoptotic T cells can also trigger the release of a set of cytokines, such as TGF-β [29], that play a key role in the pathogenesis of SSc [10]. Moreover, TGF-β induces differentiation and expansion of Treg [30] and therefore it could also have a role in determining the Treg levels in SSc. Finally, beside their effects on the immune system, since it was demonstrated that anti-ERα antibodies can act as estrogen agonists [9], we can not rule out the possibility that these antibodies could also work as pathogenic determinants exerting important modulatory effects, e.g., in the fibrotic progression [13], [14].

Several lines of evidence indicate pathogenic homologies between SSc and SLE. We refer in particular to the so-called type 1 interferon signature and the activation of the Toll-like receptor signalling [31]. With this study we provide further evidence for a similarity between SLE and SSc. In fact, serum anti-ERα antibodies were detected in patients with SLE but not in patients with other autoimmune diseases such as rheumatoid arthritis or Behçet’s disease [9]. In SSc, as well as in SLE, serum anti-ERα antibody levels were significantly related to disease activity, pointing out to the promising role of these antibodies as prognostic markers.

Overall, our study suggests that future research should be aimed at evaluating the potential therapeutic effectiveness of targeting estrogen and its receptors in SSc.
